# Absence of stress‐promoted facilitation coupled with a competition decrease in the microbiome of ephemeral saline lakes

**DOI:** 10.1002/ecy.3834

**Published:** 2022-09-22

**Authors:** Mateu Menéndez‐Serra, Vicente J. Ontiveros, Albert Barberán, Emilio O. Casamayor

**Affiliations:** ^1^ Integrative Freshwater Ecology Group Centre of Advanced Studies of Blanes (CEAB), Spanish Research Council (CSIC) Blanes Spain; ^2^ Theoretical and Computational Ecology Group Centre of Advanced Studies of Blanes (CEAB), Spanish Research Council (CSIC) Blanes Spain; ^3^ Department of Environmental Science University of Arizona Tucson Arizona USA

**Keywords:** aquatic microbiome, microbial interactions, network analysis, salt gradient, stress gradient hypothesis

## Abstract

Salinity fluctuations constitute a well‐known high stress factor strongly shaping global biological distributions and abundances. However, there is a knowledge gap regarding how increasing saline stress affects microbial biological interactions. We applied the combination of a probabilistic method for estimating significant co‐occurrences/exclusions and a conceptual framework for filtering out associations potentially linked to environmental and/or spatial factors, in a series of connected ephemeral (hyper) saline lakes. We carried out a network analysis over the full aquatic microbiome—bacteria, eukarya, and archaea—under severe salinity fluctuations. Most of the observed co‐occurrences/exclusions were potentially explained by environmental niche and/or dispersal limitation. Co‐occurrences assigned to potential biological interactions remained stable, suggesting that the salt gradient was not promoting interspecific facilitation processes. Conversely, co‐exclusions assigned to potential biological interactions decreased along the gradient both in number and network complexity, pointing to a decrease of interspecies competition as salinity increased. Overall, higher saline stress reduced microbial co‐exclusions while co‐occurrences remained stable suggesting decreasing competition coupled with lack of stress‐gradient promoted facilitation in the microbiome of ephemeral saline lakes.

## INTRODUCTION

Interactions among organisms are fundamental for the establishment and functioning of ecological communities. However, assessing the existence, magnitude, and ecological role of biological interactions is one of the most challenging goals in complex microbial communities (Faust & Raes, 2012). Microbial interactions are diverse and cover competition, facilitation, and cooperation. At the organism and population levels, competition among microbes is prevalent across different ecosystems (Ghoul & Mitri, 2016). The most generalized mechanism relies on the increase of growth rates to better compete for nutrients, space, or oxygen (Ghoul & Mitri, 2016). Positive interactions such as cooperation or facilitation have been underexplored in the microbial world and have been hypothesized to be infrequent (Foster & Bell, 2012). Likewise, stable positive interactions are only expected for organisms with limited metabolic capabilities (Machado et al., 2021) or when highly restrictive conditions prevail (Foster & Bell, 2012).

The stress gradient hypothesis (SGH) proposes that under increasing stress conditions positive interactions are more prevalent, while negative interactions are reduced as stress increases (Bertness & Callaway, 1994). This hypothesis has been supported by a wide repertoire of studies since its formulation (Bertness & Ewanchuk, 2002; Callaway et al., 2002). However, there is a current debate regarding the generality of the SGH and the role of different factors for determining expected interactions along stress gradients, fueled by some potentially contradictory results (Granda et al., 2012; Maestre & Cortina, 2004). In this context, Maestre and collaborators (Maestre et al., 2005, 2009) proposed that both the nature of the stressor along with a species' life history are key factors determining the final interaction scheme. The SGH has been mostly tested in plants, although a few studies have also found consistent results for microbes (Lawrence & Barraclough, 2016; Piccardi et al., 2019), though most of them only consider a few species in experimental laboratory incubations. Most recently, results consistent with the SGH have been found for bacteria and fungi collected from soils along an elevation gradient (Hernandez et al., 2021). One of the main reasons for the limited number of studies in natural microbial communities is due to the difficulty for assessing potential microbial interactions along wide environmental gradients.

Microbial community interactions cannot be directly observed, and indirect statistical approaches are needed to infer biological interactions (Faust & Raes, 2012). In this context, network analysis has recently gained importance to examine microbial interactions and their effects on the community structure and functioning (Barberán et al., 2012; Faust & Raes, 2012; Goberna et al., 2019; Hernandez et al., 2021). However, potential artifacts and false positive interactions have historically limited the application of correlation approaches for data collected across large environmental gradients (Connor et al., 2017) although the use of more complex statistical methods have recently tried to circumvent this limitation (Niku et al., 2019).

In the present study, we applied a combination of a probabilistic method for determining significant co‐occurrences and co‐exclusions between pairs of zero‐radius operational taxonomic units (zOTUs) (Veech, 2012) and a conceptual framework for classifying the nature of the observed co‐occurrences and co‐exclusions (Blois et al., 2014) using a microbial metacommunity studied along a wide salinity gradient. We focused on how both positive and negative potential biological interactions changed along the gradient of physiological stress linked to increasing salinity. This allowed us to test whether or not the SGH applies for complex highly dynamic microbiomes in natural environments, following previously responses observed in plant communities and simplified microbial consortia.

## METHODS

### Study site and molecular dataset

We analyzed a spatiotemporal survey on planktonic bacterial, eukaryal, and archaeal communities inhabiting the Monegros Desert lacustrine system (northeast Spain, 41°42′ N, 0°20′ W) (Menéndez‐Serra et al., 2020, 2021). Microbial community structure was assessed monthly in 14 different ephemeral shallow lakes along three calendar years, showing large salinity variations ranging from freshwater to salt saturation. The prokaryotic (bacteria and archaea) and microeukaryotic communities were analyzed by polymerase chain reaction amplification and sequencing of 16S and 18S rRNA genes, respectively. See Appendix S1 for a more detailed description of sample processing, sequencing protocols, raw sequence processing for zOTUs, taxonomic assignments, and environmental data.

### Network construction

Ecological networks were constructed based on pairwise zOTUs co‐occurrences and co‐exclusions for 116 samples. Only zOTUs with occurrences >5% (i.e., detected in at least six samples over 116) and relative abundances >0.1% in at least one sample, were considered. We applied a probabilistic model (Veech, 2012) to determine whether two zOTUs were observed together significantly more or less frequently than expected by chance (Appendix S1: Figure S1). Pairwise zOTUs associations were classified as positive (co‐occurrence), negative (co‐exclusion), or random. Link strength was defined as *s* = log(*P*_0_/*P*), where *P*_0_ is the hypothesis testing confidence level (set here at 0.01) and *P* is the observed co‐occurrence/exclusion probability, being *s* proportional to the order‐of‐magnitude difference between *P* and *P*_0_ (Martinez‐Romo et al., 2011). Significant co‐occurrences/exclusions by environmental preferences and spatial factors were tested according to the framework proposed by Blois and collaborators (Blois et al., 2014). This framework classifies significant co‐occurrence/exclusions as explained by environmental and/or spatial factors or potential interspecies interactions. We applied one‐way ANOVAs for testing significant (*p* < 0.05) environmental differences among sites where pairs of zOTUS were observed, for each of the five environmental variables, that is, salinity, temperature, pH, dissolved oxygen and redox conditions. Every environmental variable was tested independently. These variables were selected according to the previously reported effects on microbial community assembly and activity in the area (Casamayor et al., 2013; Menéndez‐Serra et al., 2020, 2021; Triadó‐Margarit et al., 2019). One‐way MANOVA was applied to test for significant (*p* < 0.05) spatial segregation or aggregation of sampling sites where pairs of zOTUs were and were not observed, respectively. Associations explained by spatial and/or environmental niche aggregation/segregation were considered as potential artifacts and therefore where not considered for network construction. Associations not explained by environment or space were considered potential biological interactions (Appendix S1: Figure S1). Subnetworks were built following the same workflow after the whole salinity gradient was previously split into three salinity ranges, defined after a consensus between ecological significance and a balanced number of selected samples. Thus, the dataset was fractionated into low (0.1%–2.4% salinity, 36 samples), intermediate (2.5%–4.9% salinity, 35 samples) and high salinity samples (5%–40%, 44 samples). For the subnetworks, a minimum occurrence threshold was not established, while the abundance threshold was retained. The source code in R for networks analysis is available at https://doi.org/10.5281/zenodo.6514358 (Menéndez‐Serra, 2022).

### Network analysis and null network model simulation

The visualization platform Gephi (Bastian et al., 2009) was used for network representation and computation of basic network parameters such as modularity, average path length, network diameter, node degree, and heterogeneity on degree distribution (Newman, 2003). The relative weight of each taxonomic group on the co‐occurrence networks was defined as the proportion of the total weighted degree accumulated by the nodes corresponding to its members. This measure was compared with its representativeness in the dataset used for making the co‐occurrence network in each case, which was defined as the proportion of the observations on the presence–absence data. Random graphs were computed by two complementary approaches to test the statistical significance of the network descriptive parameters, that is, (1) Erdös‐Rényi (Erdős & Rényi, 1960) random graph model and (2) the randomization of the presence–absence observation matrix prior to networks construction, respectively. The Erdös–Rényi random graphs were computed based on the number of nodes and the probability of interaction between nodes of the observed networks (Connor et al., 2017), preserving the average degree of the derived networks (Erdős & Rényi, 1960). Conversely, the randomization of the presence–absence matrices with the “curveball” algorithm (Strona et al., 2014) maintains species occurrences and samples richness, preserving biologic features as the distribution of generalists and specialists groups. For each network and subnetwork, 1000 random graphs were generated based on each null model approach, and its descriptive parameters were calculated to create a parameter distribution to further calculate the statistical significance (*p* < 0.05) of the observed parameters. See Appendix S1 for a detailed description of random networks construction workflow.

## RESULTS

### Pairwise co‐occurrences and co‐exclusions

**FIGURE 1:**
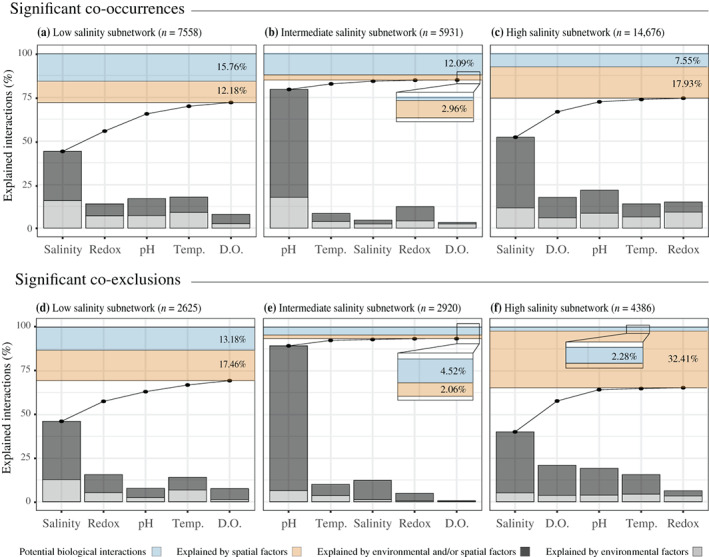
Pairwise co‐occurrences and co‐exclusions according to the framework proposed by Blois et al. (2014). For each salinity range subnetwork, we tested the potential explanation of significant co‐occurrences/exclusions looking at the differences in environmental or spatial niche. Every environmental variable was tested independently. The light gray bars indicate significant co‐occurrences/exclusions explained only by environmental factors, while dark gray bars indicate those significant zero‐radius operational taxonomic units (zOTUs) pairs that were explained simultaneously by both environmental and spatial factors. Using multiple environmental shows that species pairs can be explained by one or more variables simultaneously. We ordered these variables starting from the one that explained a higher number of associations followed by those that contributed to explain most of the proportion of associations previously unexplained by other environmental variables (black line and points), indicating a saturation of species pairs explained by the environment when adding additional variables. The species pairs explained alone by the effect of sample spatial distribution were represented above the accumulated percentage of interactions explained by the environment. Associations not explained by any environmental of the environmental variables analyzed or by spatial factors were assigned to potential biological interactions. D.O., dissolved oxygen; Temp., temperature.

Filtering based on minimum abundance and occurrence resulted in 1140 zOTUs (762 prokaryotic zOTUs and 378 eukaryal zOTUs). Significant co‐occurrences (57,795 cases detected) prevailed over co‐exclusions (26,618). Salinity explained the highest number of co‐occurrences and co‐exclusions based on niche coincidence or segregation, followed by temperature and redox condition (Appendix S1: Figure S2). In most cases, it was impossible to split the independent effect of the environment due to high spatial autocorrelation. The accumulated effect of all measured environmental variables explained 92.4% of the co‐occurrences and 98.3% of the co‐exclusions. The independent effect of space was limited (3.23% and 0.98% in co‐occurrences and co‐exclusions, respectively). Overall, the number of potential biological interactions was reduced to 2543 for co‐occurrences (4.4%) and 181 for co‐exclusions (0.68%). Subnetworks based on the three salinity ranges showed higher percentages of interaction assigned to potential biological interaction than full networks in all cases, with a decreasing trend along the salt gradient (Figure 1). Salt concentration remained as the most important parameter for the high and low salinity ranges, while pH was the environmental variable explaining most interactions at intermediate salinities.

### Network and subnetwork composition and topology

**FIGURE 2:**
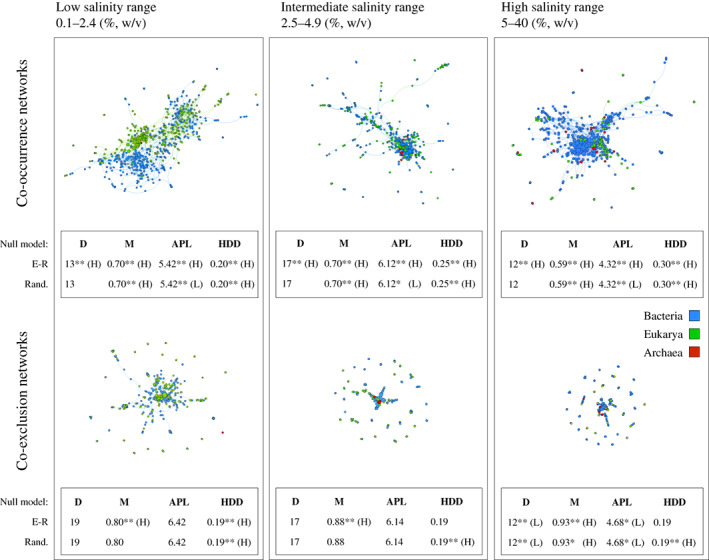
Graphical representation showing positive and negative subnetworks for each salinity range. For each subnetwork, descriptive parameters and their statistical significance according to both Erdös‐Rènyi random graphs (E‐R) and the randomized null models (Rand.) are given. D, diameter; M, modularity; APL, average path length; HDD, heterogeneity on degree distribution. L, significantly lower than expected by chance. H, significantly higher than expected by chance. Significance codes: *p < 0.05; **p < 0.001.

Network topology showed remarkable differences between positive (co‐occurrence) and negative (co‐exclusion) networks (Appendix S1: Figure S2). The co‐occurrence network showed a higher number of nodes (550 against the 221 nodes of the negative network) and links (2543 against 181), being in all cases significantly lower than that observed for the randomized null networks. Nonetheless, mean degree showed a higher connectivity between nodes than that expected by chance for the positive network, being significantly lower for the negative counterpart (Appendix S1: Figure S5). A few microbial groups were strongly overrepresented in the co‐occurrence network (Appendix S1: Figure S3). Members of the Candidate Phyla Radiation Patescibacteria such as Parcubacteria, ABY1, and Gracilibacteria showed the highest overrepresentation ratios versus their general presence in the dataset (up to ~17:1). For the salinity range subnetworks (Figure 2), significant differences were observed. Co‐exclusion networks appeared simplified as salinity increased, losing around 65% of its nodes and links. Nodes mean degree dropped from higher to lower than the observed in the randomized null models (Appendix S1: Figure S5). Heterogeneity on degree distribution, that is, the diversity in the degree of the nodes, downsized from higher than expected to random‐like values when compared to the Erdös–Rényi model, being in all cases higher than the randomized values of the null model. Network diameter and average path length, both indicative of network size and structure, progressively shifted from random‐like values to lower than expected by chance (Appendix S1: Figure S6). Conversely, co‐occurrence networks maintained their structure and complexity along the gradient. The observed significant differences found in the full network topology as compared with the null model were retained in all subnetworks. Several of the specific microbial groups that were overrepresented in the full co‐occurrence network were also overrepresented in the subnetworks, while others groups such as Spirochaetes, Lenthisphaeria, or Kathablepharidae only showed a remarkable overrepresentation within specific salinity ranges (Appendix S1: Figure S4).

## DISCUSSION

The SGH has been widely extended among plant ecologists though whether or not it can be applied to the microbial world is poorly known. Network analysis in the microbiome present along wide environmental stress gradients can unveil valuable information on how stress affects potential biological interactions and community structure. Our results showed a decrease of co‐exclusions along a gradient of increasing stress, suggesting a reduction of competition. Simultaneously, co‐occurrences remained stable, suggesting a potential lack of facilitation processes.

### Inference of potential microbial interactions and ecological interpretation

Network analysis has gained importance as a tool for understanding community assembly and functioning, complementing more classical approaches based on diversity and compositional descriptions (Barberán et al., 2012). However, current methodologies for estimating biological interactions among microbes at the community level suffer from several limitations. The robustness of correlation approaches over compositional data has been debated, and specific methodology has been developed with the aim of reducing potential artifacts (Friedman & Alm, 2012). In parallel, co‐occurrence analyses have been developed and applied (Araújo et al., 2011; Faust & Raes, 2012). Presence–absence‐based methods have proven useful (Veech, 2012) and their application over strong gradients could add more robust information than correlations. In addition to methodological advances, conceptual frameworks considering conjunctural factors to determine the potential nature of the observed associations allow to reduce the potential effect of environmental and spatial factors. Here, we found that environmental and spatial variability explained a high proportion of the observed co‐occurrences and co‐exclusions (85% and 95%, respectively) highlighting that the application of conceptual frameworks as the one proposed by Blois et al. (2014) is essential for the successful implementation of network analysis along environmental and spatial scales.

Understanding the effect that the observed patterns may have on the structure and stability of the microbial community is not straightforward. The prevalence of positive interactions has been associated with a decrease in community stability due to the increase of positive‐feedback loops (Hernandez et al., 2021). However, here we observed that positive interactions were maintained and negative interactions decreased, suggesting that community stability could remain constant along the gradient. Moreover, the high modularity observed in the system reduces the propagation of potential perturbations, limiting their effect on the community as a whole.

### The SGH in the microbial world

The SGH has fueled numerous studies in the last decades as a conceptual framework to better understand biological interactions, mostly in plant communities (He et al., 2013) and, more recently, also in microbes (Hernandez et al., 2021; Lawrence & Barraclough, 2016; Piccardi et al., 2019). Nonetheless, there is still a lack of studies assessing this question in microbial communities inhabiting natural environments where variation in stress levels occur within a limited area and can be directly quantified. In this context, saline ephemeral lakes represent a perfect scenario for assessing the role of increasing stress since salinity fluctuations occur within short times periods and distances. Incorporating the stress characteristics and organism's life history into the SGH framework (Maestre et al., 2005, 2009) improves the prediction of potential outcomes and helps further interpretations. Thus, the nature of the stress factor and the specific biology of the organisms cannot be considered independently from each other. For instance, an increase of facilitation will be only observed after, for a particular stressor, at least one member of the community has the ability to ameliorate the stress (Piccardi et al., 2019). However, the mechanistic explanation for how one given aquatic species would benefit from another under saline stress is unclear. Potentially, under salt stress a microbial facilitation mechanism could be linked to the production and excretion of compatible solutes that are used by halophilic and halotolerant organisms to maintain intracellular osmotic pressure under saline conditions (Roberts, 2005). These molecules can be either synthesized by the organism after a high energy expenditure or, alternatively, be directly incorporated from the environment (Kempf & Bremer, 1998), which substantially reduces the high bioenergetic requirements for maintaining the osmoadaptative cell machinery. However, although halophile groups relying on the “synthesis mechanism” dominate the assembly at intermediate salinities (Menéndez‐Serra et al., 2021) and take part of the retrieved co‐occurrence networks (Appendix S1: Figures S2 and S3), this strategy has not been fully described as a stable facilitation mechanism and deserves further research to establish its potential scope.

Another potential facilitation mechanism relies on intracellular parasitism. In this scenario, the parasite avoids the osmotic stress by developing its vital cycle within the host intracellular medium. Interestingly, specific microbes potentially holding this ecological strategy showed a remarkable role in the Monegros co‐occurrence network. Mostly unknown microbes of the recently proposed Candidate Phyla Radiation (CPR) (e.g., the phylum Dependentiae [Yeoh et al., 2016]) and DPANN (Castelle & Banfield, 2018) accounted for a higher proportion of interactions than expected according to their mostly modest presence in the Monegros dataset. CPR and DPANN have been hypothesized to carry out a symbiotic/parasitic lifestyle, based on a lack of essential metabolic functions in their simple genomes (Castelle et al., 2018; Yeoh et al., 2016) and direct microscopic observations in agreement with intracellular parasitism (Gong et al., 2014). Although a direct link between co‐occurrence analyses and ecology is extremely controversial, it could be still considered as a useful complementary approach. The biological nature of these potential associations, together with its perseverance along the salinity gradient, suggests an ecological behavior unaffected by the stress factor. Conversely, co‐exclusions showed a remarkable effect along the gradient, suggesting a mass shift from negative interactions to neutrality as salinity increased. An equivalent transition has been already described under increasing stress in a simplified microbial consortia (Piccardi et al., 2019) and it is most likely linked to a progressive and generalized change of the dominant strategy within the communities, moving from competition to stress tolerance (Ho et al., 2017).

### Concluding remarks

The application of indirect approaches to infer biological interactions has several methodological limitations. The main limitation is the impossibility to discern the true nature of the observed association, which limits an accurate results interpretation. Although recent studies have questioned the performance of indirect methods for inferring specific trophic associations between pairs of species (Freilich et al., 2018), when these methods are applied at larger scales, consistent patterns may arise to unveil high order interactions. Endowing observed patterns with ecological significance is controversial but also necessary for moving from describing statistical trends to generating hypotheses on the ecological processes shaping communities. Our study suggests that, in agreement with the predictions of the SGH, competition decreased along the salinity gradient potentially due to a transition in resources allocation from growth to stress tolerance. The potential lack of stress‐specific facilitation reinforces the importance of the interaction between the stress factor and the specific biology of the organisms to accurately predict potential interactions outcomes.

## CONFLICT OF INTEREST

The authors declare that they have no conflict of interest.

## Supporting information


Appendix S1

## Data Availability

Data are available from the National Center for Biotechnology Information under BioProject accession no. PRJNA429605 at https://www.ncbi.nlm.nih.gov/bioproject/PRJNA429605. Code (Menéndez‐Serra, 2022) is available in Zenodo at https://doi.org/10.5281/zenodo.6514358.
