# Supplementary material for: Absence of stress‐promoted facilitation coupled with a competition decrease in the microbiome of ephemeral saline lakes
Source: Ecology. 2022 Sep 22;103(12):e3834. doi: 10.1002/ecy.3834 (PMC10078231; doi:10.1002/ecy.3834)
Supplement: Supplementary file 1 — Appendix S1 [file ECY-103-0-s001.pdf]

Appendix S1

Supporting information for

“Absence of stress-promoted facilitation coupled with a competition decrease in the microbiome of ephemeral saline lakes”

Mateu Menéndez-Serra, Vicente J. Ontiveros, Albert Barberán and Emilio O. Casamayor

in *Ecology*

Extended methodology

Sampling scheme

The microbial dataset used in the present study contains a detailed spatio-temporal survey of bacterial, eukaryal and archaeal communities inhabiting the Monegros Desert lacustrine system (NE Spain, 41°42′N, 0°20′W), which has been already characterized in previous studies (Menéndez-Serra et al. 2020, 2021). Sampling was carried out monthly in 14 different ponds of the Monegros Desert lacustrine system along three hydroperiods. Water samples covered wide salinity variations, from freshwater (0.1 %, w/v) to near saturation (40%), both between and inside ponds due to dilution and concentration processes. Previous studies showed strong effect of salinity fluctuations on the microbial communities (Casamayor et al. 2013, Triadó-Margarit et al. 2019, Menéndez-Serra et al. 2020, 2021).

Sequencing and raw sequences processing

For DNA analyses, water samples were prefiltered in situ through a 50-µm-pore-size net to retain large zooplankton and algae, followed by a sequential filtering on 5-µm (mainly retaining nanoplanktonic and a fraction of macroplanktonic eukaryotes) and 0.2-µm (retaining bacteria and archaea) pore-size polycarbonate membranes (47- mm diameter). Microbial communities were studied by the PCR amplification and Illumina sequencing of the 16S and 18S rRNA genes. For prokaryotes, i.e. bacteria and archaea, PCR amplification of the V4 region of the 16S rRNA gene (primers set 515F-806R, Caporaso et al., 2010) and sequencing with the Illumina 2x250 MiSeq platform were carried out according to the genomic core facilities protocols and methods of the RTSF-MSU (Michigan State University, USA) (https://rtsf.natsci.msu.edu/). PCR amplification of the V9 region of the eukaryal 18S rRNA gene (primers set 1391f-EukrBr, Amaral-Zettler et al., 2009) and

sequencing with the Illumina 2x150 MiSeq platform were also carried out following the same protocols. Raw sequences were processed using the UPARSE pipeline (Edgar 2013). Sequences were merged and quality filtered (Edgar and Flyvbjerg 2015). After de-noising and chimera filtering, the UNOISE algorithm (Edgar 2013) defined Operational Taxonomic Units at 100% identity, i.e., zero-radius OTUs (zOTUs). The taxonomic assignment was carried out with SINA aligner v.1.2.11 (Pruesse et al. 2012), using the SILVA 132 reference database (Quast et al. 2013). The resulting data set contained 116 samples with 5653 prokaryotic (bacteria and archaea) zOTUs and 2658 eukaryal zOTUs. The whole gene sequence datasets were deposited to the NCBI Sequence Read Archive and are available through BioProject record ID PRJNA429605.

<u>Null network models construction</u>

In order to test the significance of the descriptive parameters of the observed networks, null network models were computed by two complementary approaches: (i) Erdös-Rényi (Erdős and Rényi 1960) random graph model and (ii) the randomization of the presence-absence observation matrix prior to the network construction process. The Erdös–Rényi random graph model G(n,p) computes graphs with a fixed number of nodes (n) and interaction probability between two nodes (p). Random graphs were created using the *sample_gnp* function on “igraph” R package (Csardi and Nepusz 2006), based on the number of nodes and the probability of interaction between nodes of the observed networks (Connor et al. 2017). For the randomization of the presence-absence matrices we implemented the “curveball” algorithm (Strona et al. 2014) which randomizes the matrices while maintaining species occurrences and samples species richness. Following matrix randomizations, network construction workflow was applied over the randomized matrices

as described in the *Network construction* subsection in the main text. This approach allows us to obtain a parameter distribution of additional networks parameters as node number, link number and mean degree, which remain fixed on the Erdös–Rényi random graphs models construction process. For both null models and each network and subnetwork, 1000 randomized networks were computed in order to create a parameters distribution to determine a p-value for the observed parameters. Observations laying outside the 95% confidence interval of the simulated distribution (p-value < 0.05) denoted a non-random assembly in the network. Null models parameters distributions and the significance of observed parameters are represented in Supplementary Figures S5 and S6.

Supplementary Figures

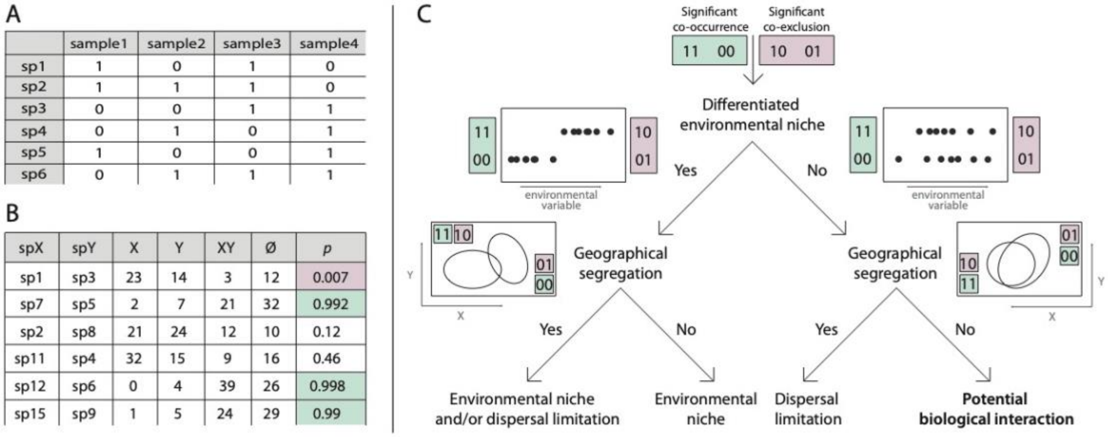
A

	sample1	sample2	sample3	sample4
sp1	1	0	1	0
sp2	1	1	1	0
sp3	0	0	1	1
sp4	0	1	0	1
sp5	1	0	0	1
sp6	0	1	1	1

B

spX	spY	X	Y	XY	Ø	p
sp1	sp3	23	14	3	12	0.007
sp7	sp5	2	7	21	32	0.992
sp2	sp8	21	24	12	10	0.12
sp11	sp4	32	15	9	16	0.46
sp12	sp6	0	4	39	26	0.998
sp15	sp9	1	5	24	29	0.99

Figure S1: Network construction workflow. From a presence/absence observation table (A), the probabilistic method proposed by Veech determines the signification of the observed co-occurrences (p) compared to a probability based on the number of samples where only one (X,Y), both (XY) or none (Ø) of the species appears (B). The observed co-occurrence can be significantly higher than expected ($p > 0.99$) evidencing a significant co-occurrence, or lower than expected ($p < 0.01$) evidencing a significant co-exclusion. Following (C), the framework proposed by Blois and collaborators allows to determine the nature of the statistically significant co-occurrences and co-exclusions. This is done after simultaneously test for each pair of species the potential differences for the selected environmental variable and the spatial distribution of samples groups. For co-occurrences analyses, differences are tested between samples where both species had been observed and samples where any of them had been observed (11,00). For co-exclusions, differences are tested between samples where species of the pair had been observed individually (10,01).

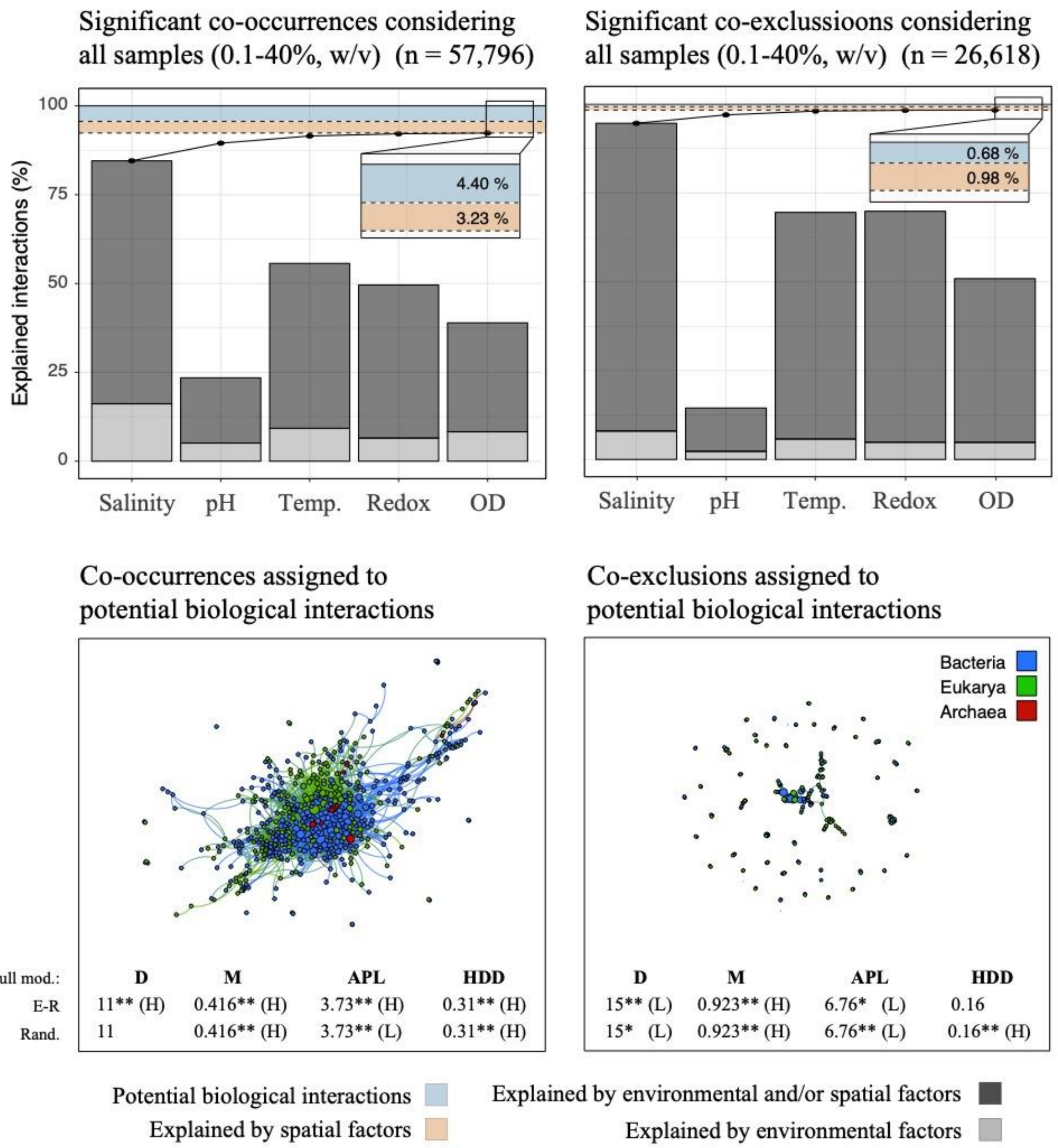

Figure S2: Pairwise co-occurrences and co-exclusions explanation according to the framework proposed by Blois and collaborators (2014) (top). Graphical representation showing positive and negative networks and the significance of its descriptive parameters according to both Erdös-Rènyi random graphs and the randomized null model. D = diameter, M = modularity, APL = average path length, HDD = heterogeneity on degree distribution. L = significantly lower than expected by chance. H = significantly higher than expected by chance. Significance codes: * p-value < 0.05; ** p-value < 0.001.

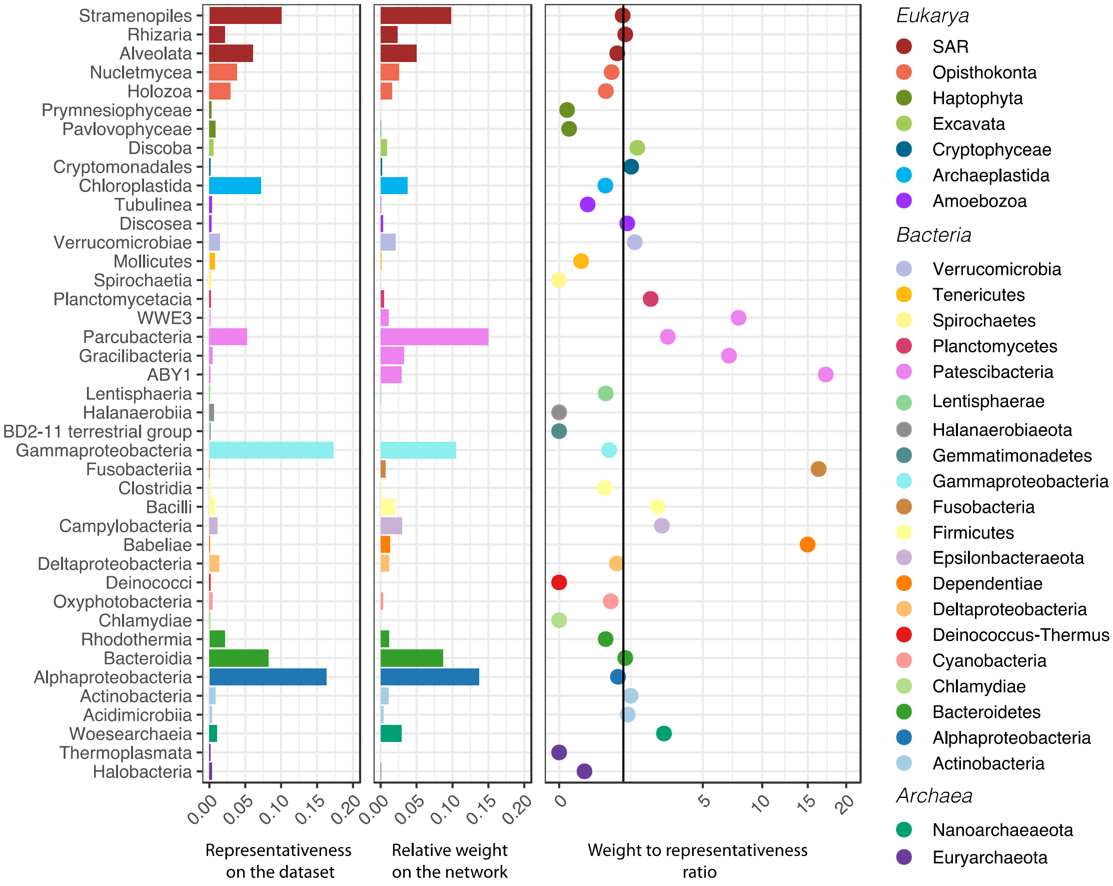

Figure S3: Representativeness on the dataset, relative weight on the co-occurrence network, and both aspects ratio for the different taxonomic groups in the complete metacommunity network. Bacteria and archaea are grouped at the class level. Eukaryotes are grouped at the L2 level of the SILVA database. Vertical black line indicates 1:1 ratio.

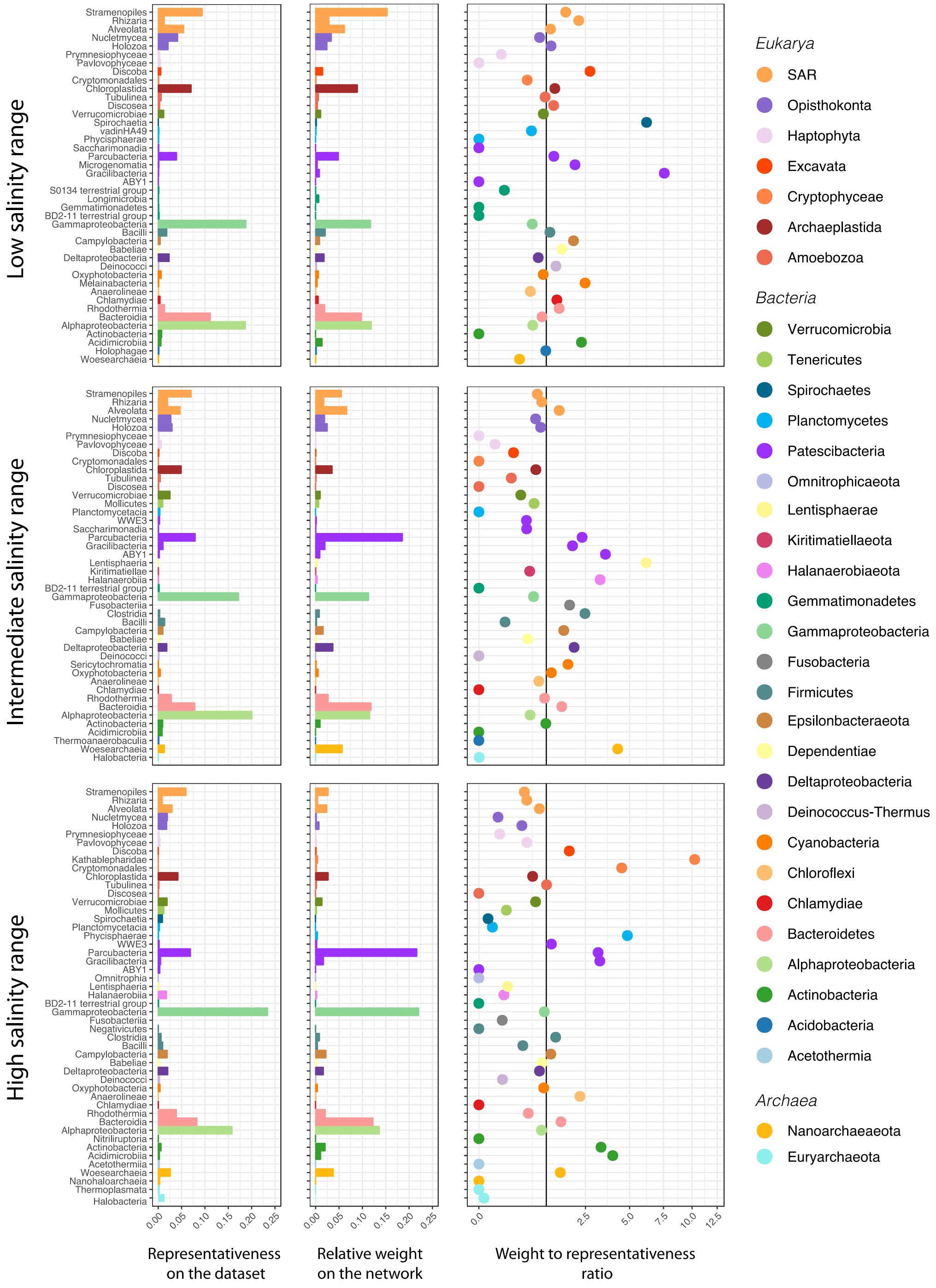

Figure S4: Representativeness on the dataset, relative weight on the co-occurrence network, and their ratio for the different taxonomic groups at the three salinity ranges. Bacteria and archaea are grouped at the class level. Eukaryotes are grouped at the L2 level of the SILVA database. Vertical black line indicates 1:1 ratio.

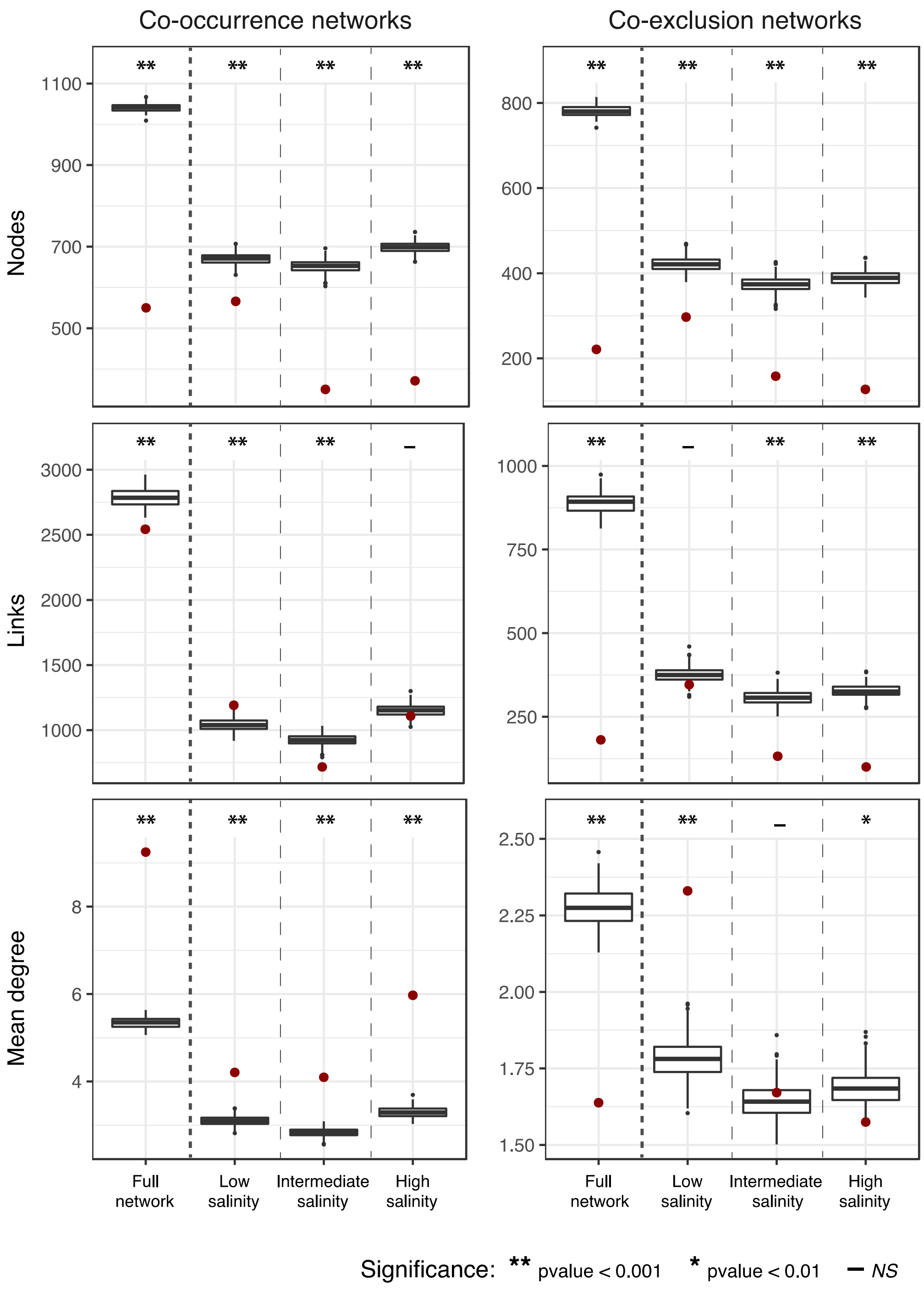

Figure S5: Boxplot representation of the network parameters exclusively obtained from the matrix randomization null model approach. Red dots show the observed parameter values in the full networks and the consecutive subnetworks.

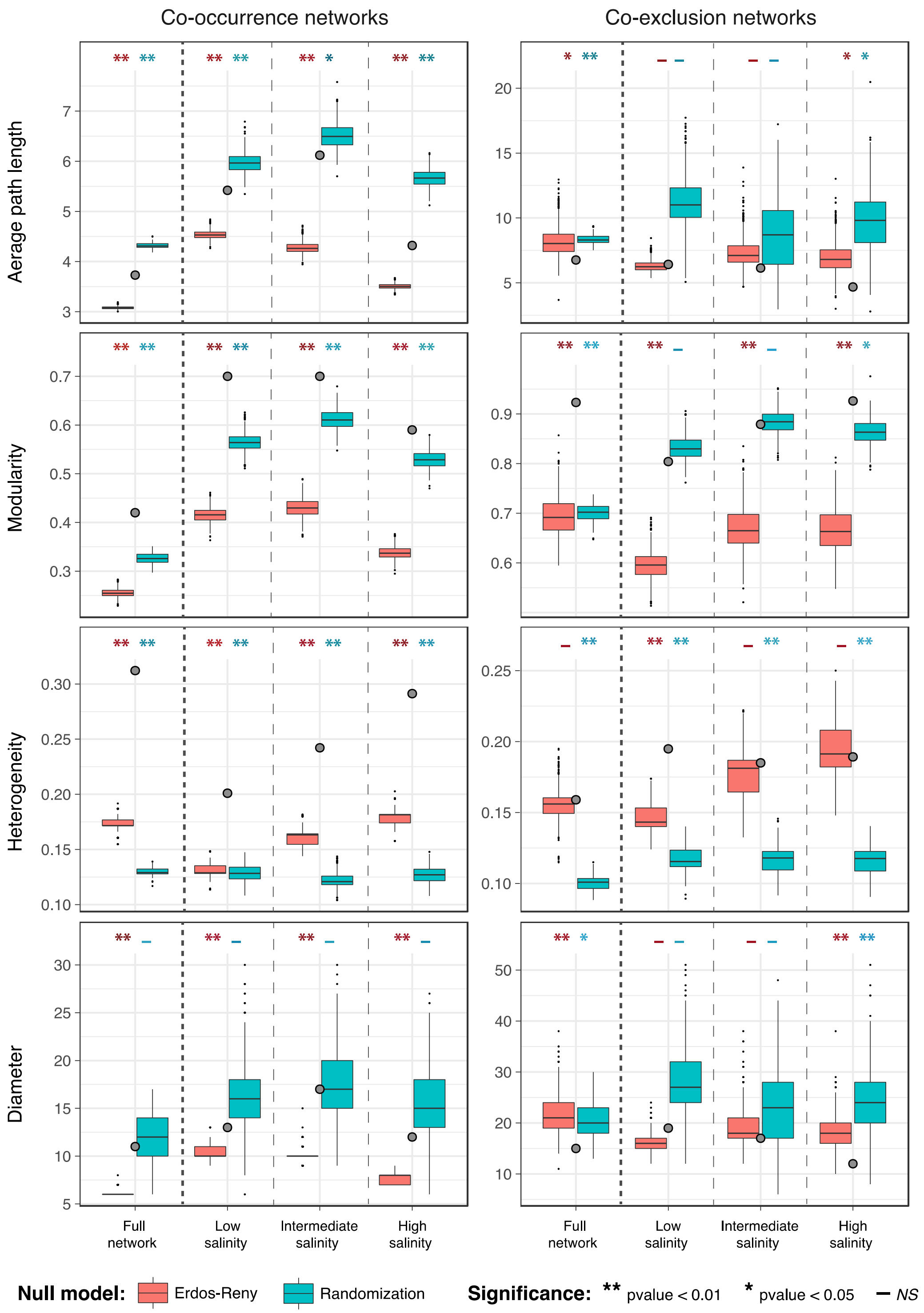

Figure S6: Boxplot representation of the networks descriptive parameters calculated for both null networks models. Grey dots represent the observed parameter values on the full networks and the consecutive subnetworks.

References

Amaral-Zettler, L. A., E. A. McCliment, H. W. Ducklow, and S. M. Huse. 2009. A Method for Studying Protistan Diversity Using Massively Parallel Sequencing of V9 Hypervariable Regions of Small-Subunit Ribosomal RNA Genes. PLOS ONE 4:e6372.

Caporaso, J. G., C. L. Lauber, W. A. Walters, D. Berg-lyons, C. A. Lozupone, P. J. Turnbaugh, N. Fierer, and R. Knight. 2010. Global patterns of 16S rRNA diversity at a depth of millions of sequences per sample. Proceedings of the National Academy of Sciences, 108:4516-4522.

Casamayor, E. O., X. Triadó-Margarit, and C. Castañeda. 2013. Microbial biodiversity in saline shallow lakes of the Monegros Desert, Spain. FEMS Microbiology Ecology 85:503–518.

Connor, N., A. Barberán, and A. Clauset. 2017. Using null models to infer microbial cooccurrence networks. PLoS ONE 12:1–23.

Csardi, G., and T. Nepusz. 2006. The igraph software package for complex network research. InterJournal Complex Sy:1695.

Edgar, R. C. 2013. UPARSE: highly accurate OTU sequences from microbial amplicon reads. Nat Meth 10:996–998.

Edgar, R. C., and H. Flyvbjerg. 2015. Error filtering, pair assembly and error correction for next-generation sequencing reads. Bioinformatics 31:3476–3482.

Erdős, P., and A. Rényi. 1960. On the Evolution of Random Graphs. Publication of the Mathematical Institute of the Hungarian Academy of Sciences. Pages 17–61.

Menéndez-Serra, M., V. Ontiveros, X. Triadó-Margarit, D. Alonso, and E. Casamayor. 2020. Dynamics and ecological distributions of the Archaea microbiome from inland

saline lakes (Monegros Desert, Spain). FEMS Microbiology Ecology, 96(3), fiaa019.

Menéndez-Serra, M., X. Triadó-Margarit, and E. O. Casamayor. 2021. Ecological and Metabolic Thresholds in the Bacterial, Protist, and Fungal Microbiome of Ephemeral Saline Lakes (Monegros Desert, Spain). Microbial Ecology, 82(4):885-896.

Pruesse, E., J. Peplies, and F. O. Glöckner. 2012. SINA: Accurate high-throughput multiple sequence alignment of ribosomal RNA genes. Bioinformatics 28:1823–1829.

Quast, C., E. Pruesse, P. Yilmaz, J. Gerken, T. Schweer, P. Yarza, J. Peplies, and F. O. Glöckner. 2013. The SILVA ribosomal RNA gene database project: improved data processing and web-based tools. Nucleic acids research 41:D590-6.

Strona, G., D. Nappo, F. Boccacci, S. Fattorini, and J. San-Miguel-Ayanz. 2014. A fast and unbiased procedure to randomize ecological binary matrices with fixed row and column totals. Nature Communications 5:4114.

Triadó-Margarit, X., J. A. Capitán, M. Menéndez-Serra, R. Ortiz-Álvarez, V. J. Ontiveros, E. O. Casamayor, and D. Alonso. 2019. A Randomized Trait Community Clustering approach to unveil consistent environmental thresholds in community assembly. The ISME Journal, 13:2681-2689.
